# Diagnostic approach and grading scheme for canine allergic conjunctivitis

**DOI:** 10.1186/s12917-022-03561-5

**Published:** 2023-02-03

**Authors:** Esmeralda Delgado, Érica Gomes, Solange Gil, Ana Mafalda Lourenço

**Affiliations:** 1 CIISA – Centre for Interdisciplinary Research in Animal Health, Faculty of Veterinary Medicine, University of Lisbon, Lisbon, Portugal; 2 Associate Laboratory for Animal and Veterinary Sciences (AL4AnimalS), Vila Real, Portugal; 3 HEV, Hospital Escolar Veterinário, Faculty of Veterinary Medicine, University of Lisbon, Lisbon, Portugal

**Keywords:** Canine allergic conjunctivitis, Diagnostic criteria, Clinical score

## Abstract

**Background:**

In humans, allergic conjunctivitis is a well described disease. In contrast, allergic conjunctivitis has not received much attention from the veterinary community so far. Canine allergic conjunctivitis (cAC) is one of the possible manifestations associated with canine atopic dermatitis (cAD), being often underdiagnosed and undertreated. Our aim is to contribute to disease characterization and clinical stagingfor cAC severity.

**Results:**

A retrospective observational study including 122 dogs that underwent a complete ophthalmological and dermatological examinations and diagnosed with allergic conjunctivitis was conducted. A total of six ophthalmic clinical signs were considered for disease characterization and clinical staging: conjunctival hyperemia, chemosis, ocular pruritus, epiphora, seromucoid to mucopurulent discharge and keratitis, classified from 0 (absent) to 3 (severe). Scores comprised between 1–5 were considered mild, 6–10 moderate and 11–18 severe. The majority of dogs (64%) presented with moderate cAC followed by 24% of mild stages and only 12% of severe presentations. The severity of allergic conjunctivitis was not correlated to sex or age at the time of diagnosis and all presented with a bilateral form of the disease. Chemosis (84%), hyperemia (83%) and ocular pruritus (79%) was observed in 55% of the cases. Seromucoid to mucopurulent discharge (62%) and epiphora (69%) were less frequent, with keratitis being the least encountered clinical sign (15%). The degree of keratitis showed a positive correlation with both severity and chronicity of cAC (rho = 0.21–0.29, *p* ≤ 0.02)). Severity of cAD and cAD were not significantly correlated (*p*-value = 0.4).

**Discussion and conclusion:**

The triad hyperemia, chemosis and ocular pruritus, already known in human medicine to be a reliable way of diagnosing human allergic conjunctivitis, also proved to be important in cAC Mild forms of the disease may pass unnoticed, ocular pruritus being hard to assess in canine patients.The proposed standardized diagnostic approach and novel grading scheme for cAC may be of value for both veterinary ophthalmologists and dermatologists, as well as general practitioners.

## Introduction

In humans, allergic conjunctivitis is a well described disease. One third of the human population is affected by some form of allergy, and 40% to 60% of this population has ocular manifestations [1]. Allergic conjunctivitis is the most common form of ocular allergy in people [2] and is often associated with allergic rhinitis. Allergic conjunctivitis is known to alter the quality of life in affected patients, causing ocular discomfort, changes in vision, and making usual daily tasks difficult [1]. In fact, red itchy eyes were often consisdered ‘extremely bothersome’ in many allergic humans, similar to nasal congestion [3].

In contrast, allergic conjunctivitis has not received much attention from the veterinary community so far. In dogs with mild allergic conjunctivitis symptoms, the disease may pass unnoticed for a long period of time. Squinting, photophobia or ocular pruritus may be underappreciated by pet owners, confounded with what could be perceived as ‘normal canine behavior’ or simply a manifestation of facial pruritus from canine atopic dermatitis. Presumptive diagnosis of canine allergic conjunctivitis (cAC) is made by exclusion of other causes of conjunctivitis, consistent history, positive results on allergy testing (serology or intradermal tests), and/or conjunctival provocation tests [4]. Our research group previously showed that the experience of the veterinarian is crucial in the correct diagnosis of allergic conjunctivitis in dogs [4], and concluded there was a critical need to establish standardised criteria for proper disease diagnosis among general practitioners and veterinarians of varied speciality training [4]. Similar to published criteria in human patients with allergic conjunctivitis [5–7], standardized criteria in dogs would facilitate timely diagnosis and clinical staging of cAC, and pinpoint the best treatment options to improve the wellbeing of canine patients.

Supported by human literature and the authors’ clinical experience, the present work proposes a standardized approach to disease characterization and clinical staging by considering six different clinical signs. Using this approach, three disease severities (mild, moderate and severe) emerged. Standardized tools will undoubtedly help general practitioners, veterinary dermatologists and veterinary ophthalmologitis to reduce time consuming diagnostics, help in the monitoring of clinical evolution, and assist in tailoring patients therapy.

## Methods

A prospective observational study including 122 dogs was conducted. Patients included in the study were admitted to the Ophthalmology or Dermatology services of a Veterinary Teaching Hospital with a clinical history compatible with cAC. The study was approved by the Ethics Committee (ref 028/2020) of the Faculty of Veterinary Medicine, University of Lisbon, and owners gave written consent for the use of clinical data related to their pets.

### Study population

A previously assembled clinical database, containing 122 cases of cAC fully characterized between 2013 and 2019, with both complete ophthalmological and dermatological examinations, was used for the purpose of this study. The selected dogs showed no other changes in the ophthalmological examination, except for the suspicious signs of canine allergic conjunctivitis, and no other dermatological diseases besides atopic dermatitis. No age, sex or breed restrictions were established.

Inclusion criteria were: a full ophthalmological examination in the description of the animal's clinical records, including the characterization and grading of the cAC based on the six different clinical signs, a description of the prescribed treatment and a complete dermatological examination including the classification of the lesional score of atopic dermatitis using the internationally accepted Canine Atopic Dermatitis Extent and Severity Index (CADESI)-03 or CADESI-04 scale [8].

### Ophthalmic diagnostic evaluation

All the animals underwent a complete ophthalmic examination in order to rule out the main differential diagnoses, such as 'keratoconjunctivitis sicca' (quantitative deficiency), corneal ulceration or conjunctivitis due to other etiologies such as eyelid conformational changes or distichia/ectopic cilia, contact with irritating substances or foreign bodies, neoplasia or intraocular diseases, and reach a final diagnosis of cAC through exclusion of other causes and compatible clinical examination.

Briefly:

- Inspection of both eyes from a distance, including the eyelids and the remaining periocular region;
- Evaluation of the menace response bilaterally;
- Evaluation of the palpebral, corneal and pupillary light reflexes bilaterally;
- Schirmer tear test-1 (STT-1; Schirmer tear test strips®, Eickemeyer, Sunbury-on-thames, United Kingdom). Normal aqueous tear production was considered to be in the range of 15—25 mm/min [9];
- Measurement of the intraocular pressure (IOP) using rebound tonometry TonoVet® (Kruuse, Denmark). The pressures were considered normal when the values were between 15—25 mmHg [9];
- Slit lamp biomicroscopy (Kowa SL-15, Tokyo, Japan) to rule out any changes in the anterior chamber, iris or lens and to rule out changes in the conjunctiva other than hyperemia/chemosis (such as foreign body, tumor etc.)
- Tyndal effect to rule out intraocular inflammation/uveitis;
- Funduscopy using modified indirect ophthalmoscopy (PanOptic®, Ophtalmoscope. Welch Allyn, Skaneateles Falls, USA) to rule out posterior inflammation or other pathologies of the posterior ocular segment;
- Fluorescein test (Fluorescein®; Haag-Streit International, Köniz, Switzerland) to rule out the presence of corneal ulcers and confirm the patency of the nasolacrimal outflow system through the Jones dye test.

A total of six clinical signs were considered for disease characterization and clinical staging: conjunctival hyperemia, chemosis, ocular pruritus, epiphora, seromucoid to mucopurulent discharge and keratitis based on previous studies by our group [4]. These clinical signs were graded from 0 (absent) to 3 (severe) according to their severity, with the aid of slit lamp biomicroscopy for conjunctival and corneal changes.

Pruritus was rated based on asking the owners about frequency/severity of ocular pruritus at home, based on observation of the dog itching the periocular area during the exam and by observation of the eventual lesions on the periocular skin There is an inherent difficulty in evaluating this sign in dogs, being largely dependent on the clinical history obtained from the owners and their perception of itching-related behaviours.

### Dermatological evaluation

All dogs underwent a complete dermatological examination and classification of the severity of atopic dermatitis (AD) using the Canine Atopic Dermatitis Extent Severity Index (CADESI). Two versions were used (03 and 04), according to the current version at the time of visit. In order to allow for data uniformization, all CADESI quantitative values were converted into a qualitative scale (mild, moderate, severe or in remission).

Of note, a large portion of the study population (110 out of 122) also received an extra skin score for the head region in order to assess skin involvement of the periocular region. Although this region is not included in the CADESI scale, it is considered relevant in dogs with allergic conjunctivitis due to the potential presence of ocular pruritus.

No anesthesia procedure was followed on animals for their dermatological or ophthalmic evaluations.

### Grading score for cAC

Six clinical signs were considered for cAC staging: conjunctival hyperemia, chemosis, ocular pruritus, epiphora, seromucoid to mucopurulent discharge and keratitis.

For each clinical sign a score ranging from 0 (absent) to 3 (severe) was assigned. A total score comprising the six results was assessed for each eye, resulting in a quantitative value between 1 and 18.

Based on this scoring result, we averaged the score from the right and left eye, in order to get a single score per animal for cAC severity. Based on the analysis of the scores obtained and the correspondent clinical scenarios, a severity grading scheme was elaborated. Scores comprised between 1–5 were considered mild, between 6–10 moderate and between 11-18 severe.

### Data analysis

Data was analysed using Excel and R Commander version 4.0.0. Mean and standard deviation of numerical variables were calculated, and the presence and severity of clinical signs were recorded.

Distribution of animals by sex (1-sample proportion test) and a possible relation of sex with CADESI's qualitative grade and clinical score for cAC was evaluated using the Chi-square test. The Shapiro–Wilk test was used to assess normal distribution of each outcome; parametric and non-parametric statistical tests were used to assess normally distributed and non-normally distributed outcomes, respectively. The influence of age on the severity of cAC and cAD was assessed using Kruskal–Wallis tests. Using Chi-square and ANOVA tests, the relationship between the severity of the cAC severity grade and CADESI classification was evaluated. Spearman’s correlation tests were used to assess for the correlation between ocular pruritus and score of the dermatological lesions in the head regions, as well as the correlations between the overall eye score (average of both eyes) and the scores of chemosis, hyperemia, keratitis, and ocular pruritus. Results were considered statistically significant when *p* < 0.05, thereby a 95% confidence interval was used.

## Results

### Caracterization of the studied population

Our sample included 58 females (47.5%) and 64 males (52.5%), showing no statistically significant sex predisposition for this disease (*p*-value = 0.587). Concerning age, there was a very broad range of patient distribution in our study population. Patients were aged between 10 months and 13 years, presenting an average ± standard deviation age of 4.80 ± 2.94 years. No correlation between age and severity of clinical signs of both cAC (*p*-value = 0.8) or AD was found (*p*-value = 0.9).

A total of 27 breeds were represented, mixed breed being the most common one. The number of dogs of each breed within the studied population is represented in Table 1.

**Table 1:** Number of individuals of each breed within the studied population

Breed	Number of dogs
Mixed breed	29
Labrador Retriever	16
French Bulldog, Shih Tzu	9
English Bulldog	7
Cocker Spaniel, Weimaraner, Yorkshire Terrier	6
Golden Retriever, Pug	4
Boxer	3
American Staffordshire, Cavalier King Charles, Fox Terrier, German Shepherd Dog, Portuguese Water Dog	2
Basset Hound, Beagle, Bordeaux Dogue, Border Collie, Bullmastif, Pomeranian Dog, Pitbull, Pinsher, Portuguese Pointer, Rafeiro Alentejano, Springer Spaniel, West Highland White Terrier	1
**Total sample size**	**122**

### Ophthalmic examination findings

**Fig. 1:**
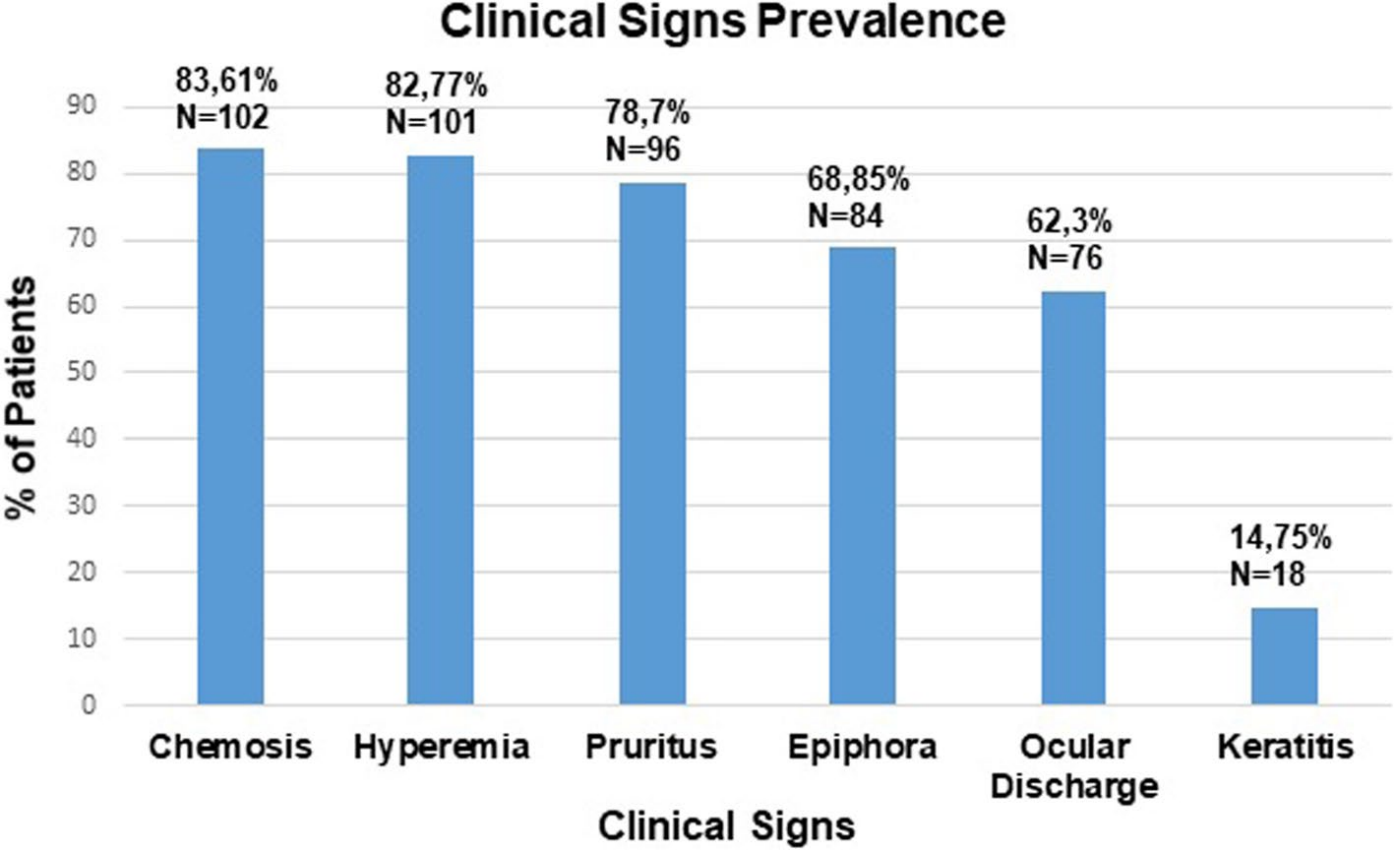
Prevalence of allergic conjunctivitis clinical signs within the studied population (Ocular discharge corresponds to seromucoid to mucopurulent discharge)

**Fig. 2:**
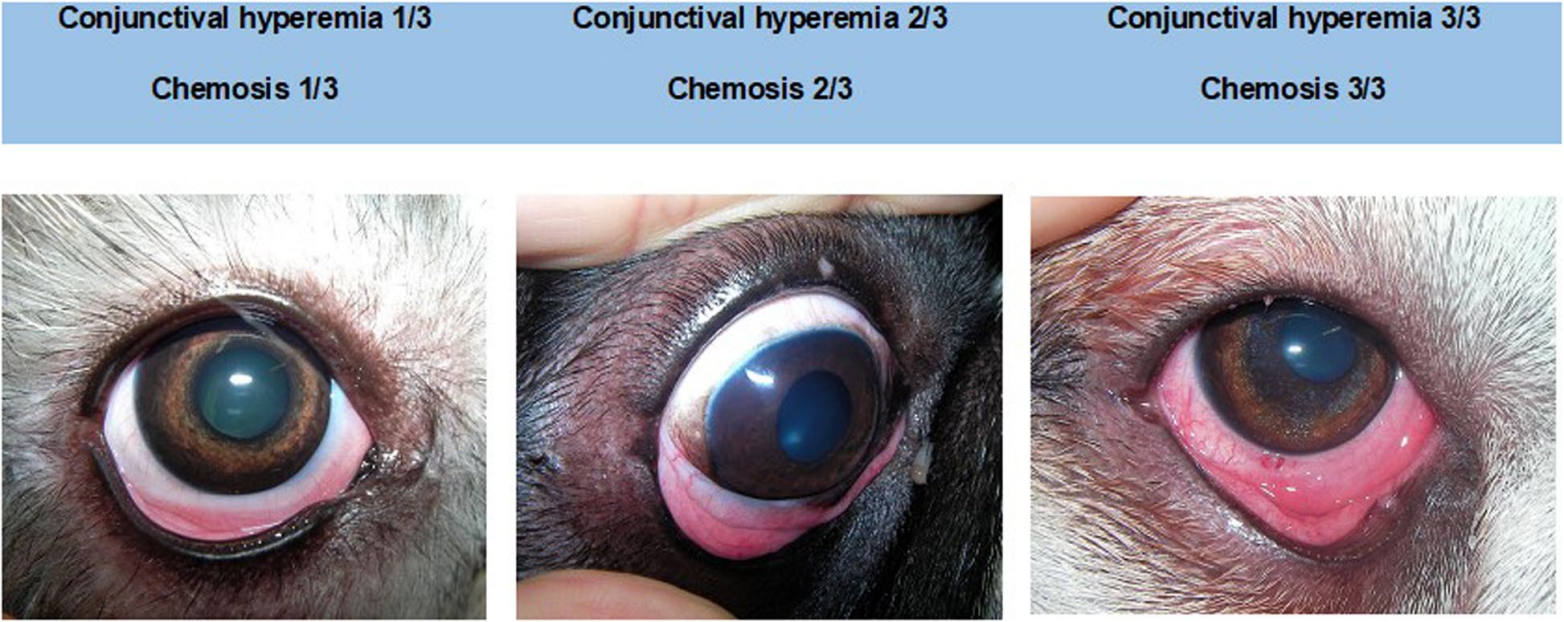
Example of clinical cases that illustrate the three different degrees of chemosis and conjunctival hyperemia, the two most prevalent clinical signs of cAC that can help clinicians stage disease severity

All the animals presented normal STT-1 and IOP values. Furthermore, Tyndall effect was negative and no signs of intraocular inflammation were detected on ophthalmic examination in any patient. Funduscopy was normal in all cases. Concerning the clinical characterization of cAC, the most prevalent clinical signs were chemosis (84%), followed by conjunctival hyperemia (83%) and ocular pruritus (79%). The three most common clinical signs of this disease (chemosis, hyperemia and ocular pruritus) were all present in 55% of the canine patients; in other words, more than half of cAC patients presented with a triad of chemosis, hyperemia and ocular pruritus (Figs. 1 and 2).

**Fig. 3:**
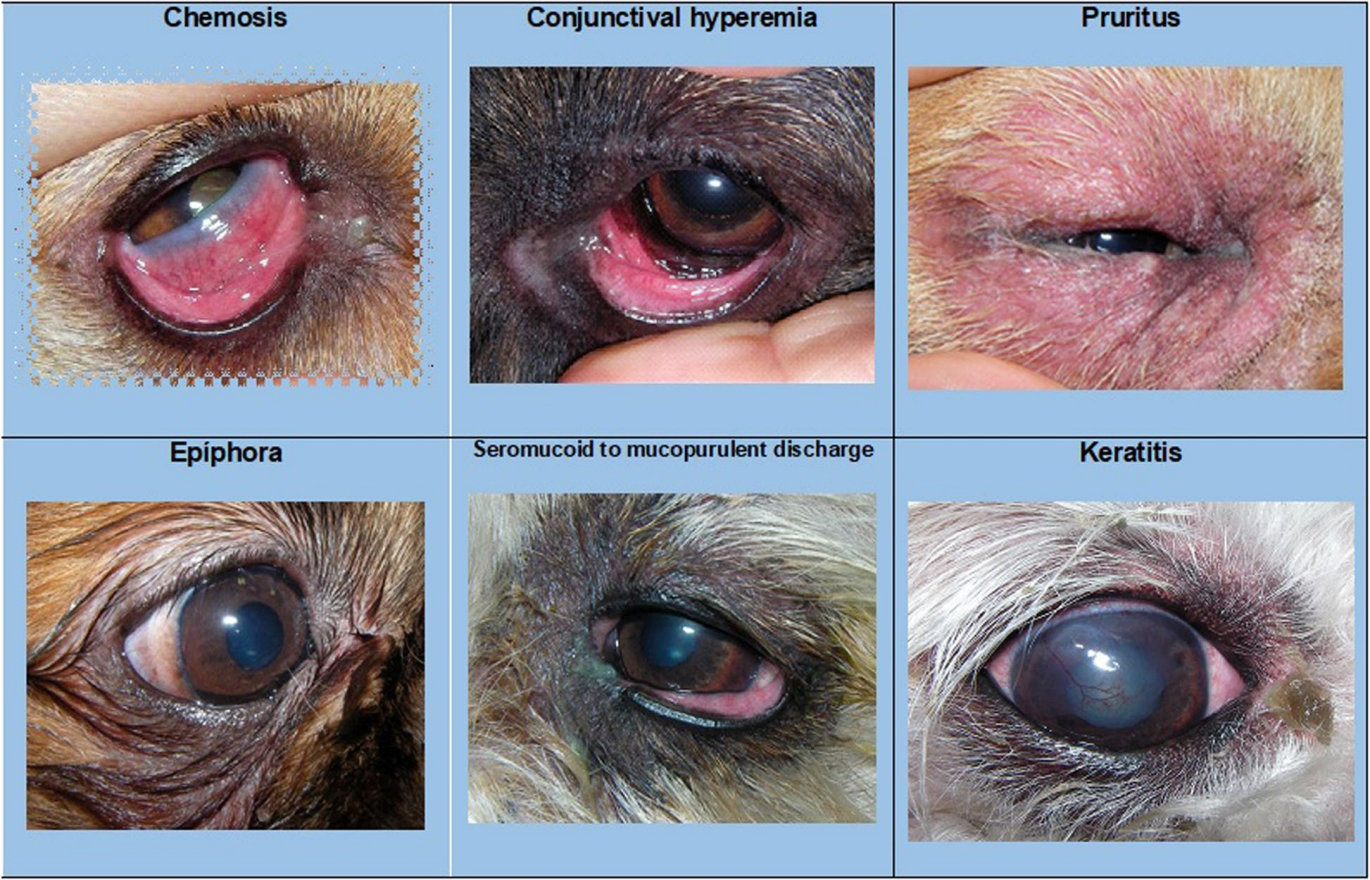
Photograph of an example of each characteristic clinical sign of allergic conjunctivitis

Epiphora (69%) and seromucoid to mucopurulent discharge (62.%) were less frequent, with keratitis being the least frequent clinical sign (15%). Keratitis score showed a positive correlation with both severity and chronicity of cAC (OD: *p*-value = 0.001 and rho = 0.29; OS: *p*-value = 0.02 and rho = 0.21). Figure 3 provides an example of each of the six clinical signs that characterize this disease.

**Fig. 4:**
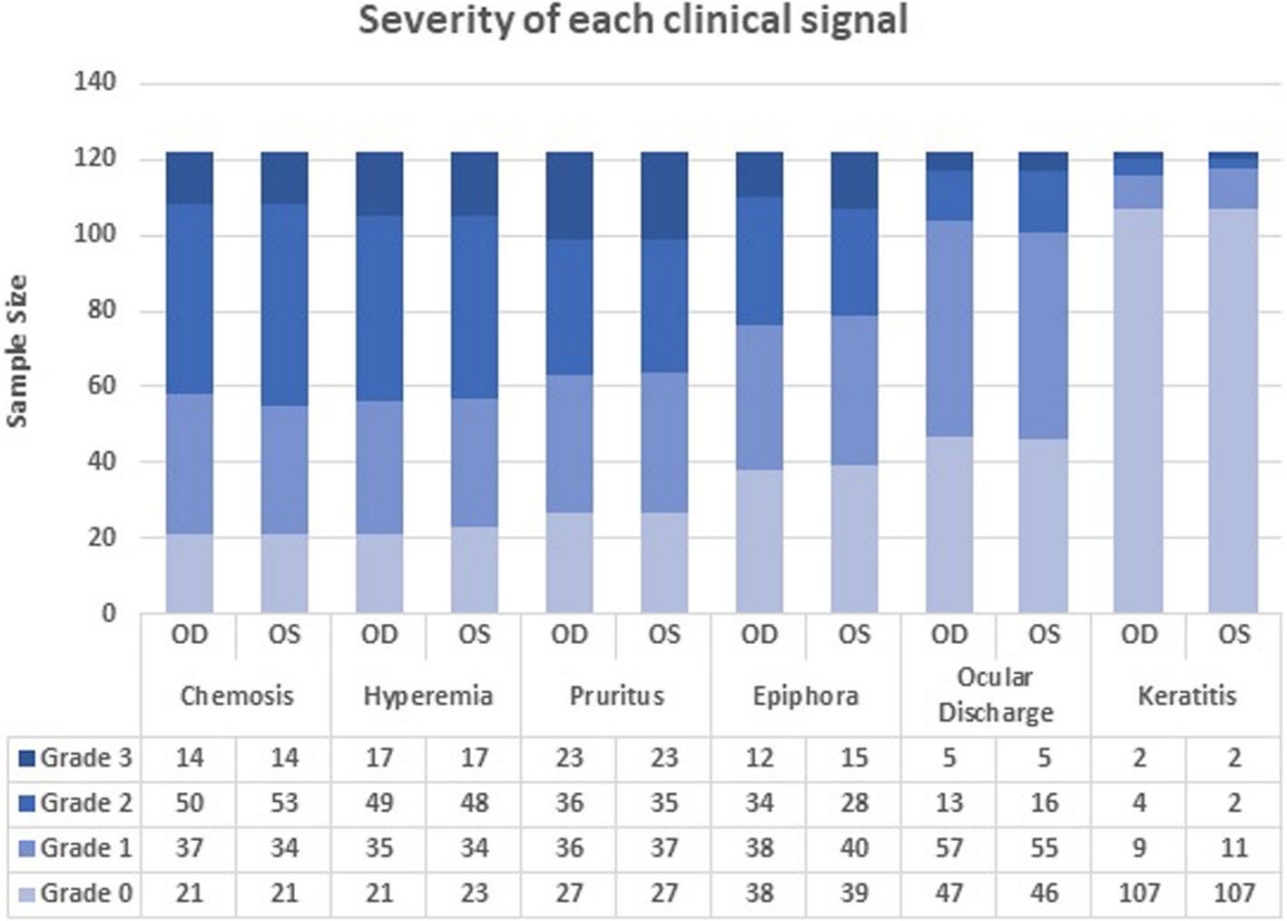
Severity grading of the six clinical signs in the right (OD) and left (OS) eyes of dogs diagnosed with allergic conjunctivitis (n = 122). (Ocular discharge corresponds to seromucoid to mucopurulent discharge.)

The distribution of the severity of each clinical sign in the right (OD) and in the left eye (OS) within the studied population is represented in Fig. 4.

Mean ± standard deviation scores for cAC were 6.54 ± 2.90 in the left eyes and 6.57 ± 2.90 in the right eyes, that is, 6.55 ± 2.90 when averaging both sides. All cases (100%) presented with bilateral disease that either had the same severity in both eyes (symmetrical, 73%) or was considered assymetrical (27% dogs). In the assymetrical presentations, the difference between the quantitative scores for both eyes was relatively low, on average 1.40.

### Dermatologic examination findings

CADESI-03 or 04 values obtained according to the degree of cAD signs were converted into a qualitative scale (mild, moderate, severe or in remission). After analysis of the results, the distribution of cAD severity was as follows: 48/122 of the dogs presented with a mild AD, 49/122 moderate, 18/122 severe and 7/122 dogs were in remission of their atopy. Overall, the majority of dogs included in our study presented with both cAC and cAD symptons (115/122, 94%).

The severity of cAD signs was not significantly associated with sex (*p*-value = 0.5) or age (*p*-value = 0.9) on initial presentation.

A total of 110 out of the 122 dogs also had a positive score attributed to the head region (sum of corresponding CADESI values), which took into account the local severity of erythema, lichenification, alopecia and excoriations. The average value of the lesions present in this region was 14.45 ± 14.07. The degree of ocular pruritus was not significantly correlated with the head score (*p*-values ≥ 0.64).

The severity of atopic dermatitis lesions was not significantly associated with the clinical score for allergic conjunctivitis (*p*-value = 0.4).

### Grading scores of cAC

**Fig. 5:**
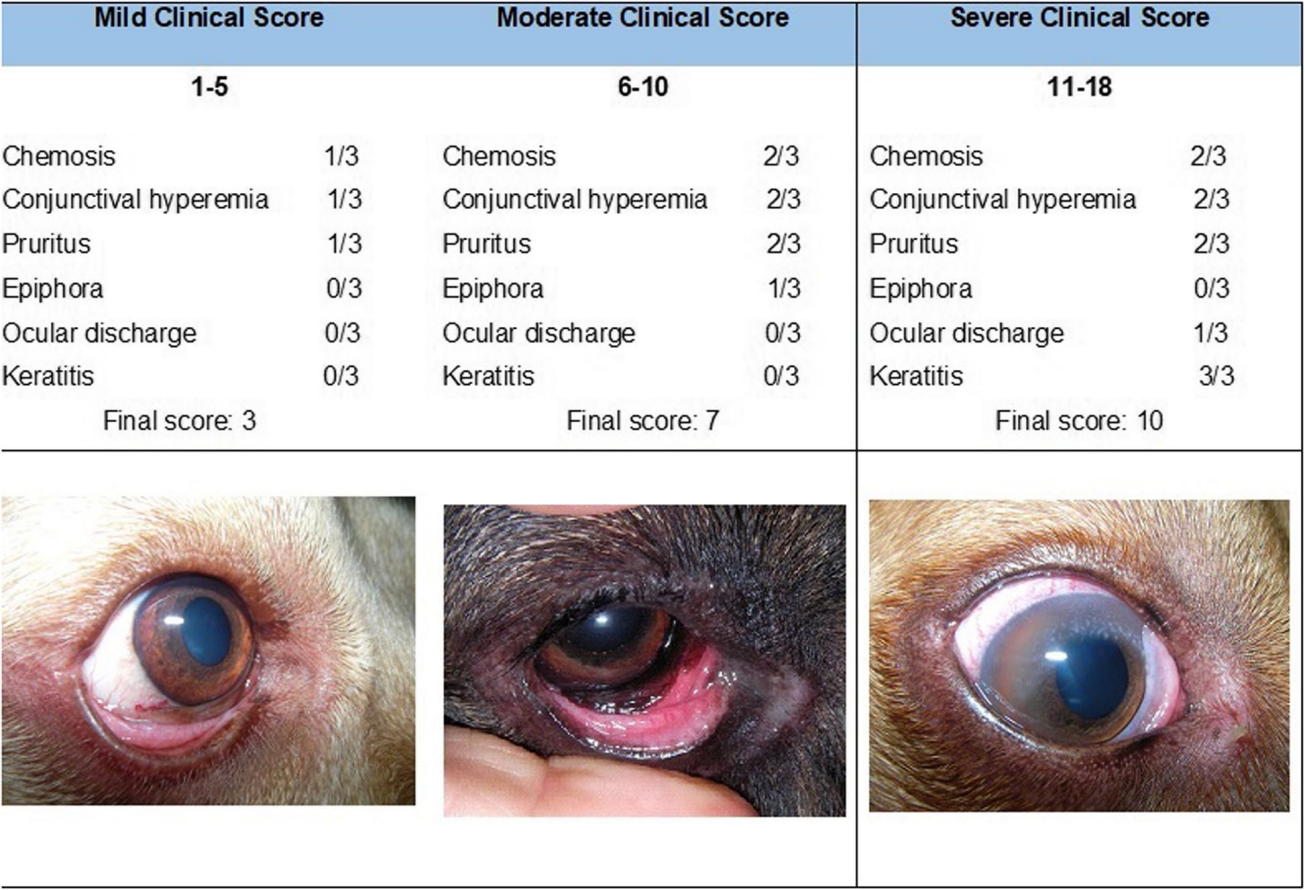
Proposed Grading Score for the severity of cAC with clinical examples of each grade. (Ocular discharge corresponds to seromucoid to mucopurulent discharge.)

Moderate disease was the most common form of cAC presentation, comprising a total of 64% (78/122) of the cases, followed by 24% (29/122) of mild stages and only 12% (15/122) of severe presentations. The proposed grading scheme with a clinical example of each severity grade can be found in Fig. 5.

Although the majority of the studied population presented with concomitant cAD (115/122, 94%), severity of both diseases was not significantly associated (*p*-value = 0.4).

Additionally, the severity of the clinical presentation of cAC was not significantly associated with sex (*p*-value = 0.06) or animals’ age (*p*-value = 0.8) at the time of diagnosis.

The scores of the three clinical signs that composed the triad were found to be positively correlated with the final quantitative score for cAC (ocular pruritus OD: *p*-value = 1e-14 and rho = 0. 63; ocular pruritus OS: *p*-value = 2.3e-15 and rho = 0.64; chemosis OD: *p*-value < 2.2e-16 and rho = 0.67; chemosis OS: *p*-value < 2.2e-16 and rho = 0.70; hyperemia OD: *p*-value = 4.87e-10 and rho = 0.53; hyperemia OS: *p*-value = 4.46e-10 and rho = 0.53). This finding means that intense iocular pruritus, high degree of chemosis and conjunctival hyperemia were more likely to be present in moderate/severe cases compared to the other clinical signs.

Despite its low incidence, the degree of corneal involvement/keratitis was more frequent in the most severe cases, being also positively correlated with the quantitative score for cAC (OD: *p*-value = 0.001 and rho = 0.29; OS: *p*-value = 0.02 and rho = 0.21).

## Discussion

In spite of being a common disease and in contrast to humans, there is very little information about allergic conjunctivitis in the veterinary literature. In fact, cAC is one of the main differential diagnoses for a "red eye". The allergic response can occur without other allergic manifestations, but it is more common in conjunction with AD [4]. Our research group has previously reported hat nearly 60% of dogs with AD had concomitant allergic conjunctivitis [4]. Clinical signs in these dogs included conjunctival hyperemia, chemosis, ocular pruritus, epiphora, seromucoid to mucopurulent discharge and keratitis [4].

In human medicine the percentage of cases with allergic conjunctivitis diagnosed in atopic patients depends largely on the medical specialty, and ophthalmologists are more qualified to appreciate more subtle clinical signs and diagnose milder forms of the disease [10]. A canine study also found this discrepancy between the veterinary medical specialties, raising the suspicion that canine allergic conjunctivitis is also underdiagnosed and undertreated in general practice [4].

The triad of hyperemia, chemosis and ocular pruritus, already known in human medicine to be a reliable way of describing human allergic conjunctivitis [11], also proved to be important in this canine study, correlating with the final score and therefore with the severity of the disease. More than half of the studied dogs presented with this triad, which reinforces its importance in the diagnosis of cAC. These clinical signs are caused by early phase allergy mediators, of which histamine is the most important [1, 11], and are referred in human medicine as a fairly reliable way of describing allergic conjunctivitis [5]. Thus, evaluation of these signs is fundamental for characterization of the disease.

Pruritus is classified in human medicine as the most characteristic clinical sign of allergic conjunctivitis [12, 13]. In our study, although this clinical sign was the one most often classified with the maximum degree of severity (degree 3), when compared to the other clinical signs, 21% of animals apparently did not manifest it. This percentage may result from the inherent difficulty in evaluating this sign in dogs, being largely dependent on the clinical history obtained from the owners and their perception of itching-related behaviours.

Nearly 60% of the patients showed epiphora and seromucoid to mucopurulent discharge, usually in mild presentations. These signs are also described in the literature as typical of the disease, especially if associated with the triad [4, 14, 15]. Keratitis was the least frequent clinical sign (15%) and, when present, it was usually mild, which is in accordance with the literature [4]. However, there was a positive correlation between the degree of corneal involvement and disease severity. In humans, corneal involvement is characteristic of severe and chronic forms of the disease, such as atopic keratoconjunctivitis and vernal keratoconjunctivitis. Generally, in the milder forms such as seasonal/intermittent allergic conjunctivitis and perennial/persistent allergic conjunctivitis, there is no corneal involvement [5, 12]. Therefore, it seems logical that dogs with keratitis are those presenting with the most severe and chronic forms of cAC. We believe that the etiology for corneal lesions in cAC is multifactorial, being due to the chronic inflammatory reaction associated with liberation of cytokines and other mediators and also to changes associated with the tear film quality. So, ocular lubricants will probably help in several ways, not only by acting as a physical barrier for allergens but also by stabilizing the tear film.

Our proposed score for allergic conjunctivitis has considered 3 levels of severity corresponding to mild, moderate and severe, similar to what is described in human medicine [5–7]. It was based only on the assessment of clinical signs existing at the time of the ophthalmological examination, given the inability of assessing symptoms or changes in performing daily tasks in dogs, compared to humans [5–7]. For the same reasons, the persistence or intermittence of clinical signs was also not considered in dogs, contrary to some human scores [5].

The severity of allergic conjunctivitis was not correlated to sex or age at the time of diagnosis; the same appears to be true in dogs with atopic dermatitis [16], although there has been some inconsistency in results regarding the possible absence of sex predisposition [16]. In our study population, pure-bred dogs predominated (93 /122 dogs), distributed across 27 breeds. This breed distribution is consistent with what has been described before for cAD [8].

The intervals for each clinical score were defined based on the observation of the distribution of clinical signs and clinical experience of the reseachers. The triad (hyperemia, chemosis and ocular pruritus) is generally present in the majority of diagnosed cases. If each of these clinical signs obtains a mild grade (grade 1), even if eventually one of the signs is a little more severe obtaining a grade 2, we obtain a score up to 4 points (mild). When the triad is given a moderate score (grade 2) and the presence of epiphora and, less frequently, seromucoid to mucopurulent discharge is present in slight (1) to moderate (2) degrees, the final score usually reaches a value between 5 and 10 points. When the cornea is involved and the triad is more severe, and there may be epiphora and/or seromucoid to mucopurulent discharge present, we get scores that exceed 10 points, corresponding to the severe presentation cases.

The moderate clinical cases of cAC were the most common form of presentation of the disease, comprising a total of 64% of the cases, followed by 24% of mild stages and only 12% of severe presentations. This could indicate that the disease in dogs has more severe manifestations than in human patients, where mild forms are the most frequent presentations [17], or that dogs are first examined by the veterinarian at a much later time in disease progression. On the other hand, it also raises the hypothesis that the mild forms of the disease may pass unnoticed, as the animal does not verbalize symptoms of eye discomfort and often the ocular pruritus manifests itself through non-specific behaviours such as head rubbing on objects (e.g., sofas, carpets, owners' legs).

All dogs presented with a bilateral form of the disease, which is also the case in humans [17]. The vast majority of cases were symmetrical (73%), and the asymmetric cases showed only discrete differences between the clinical scores of both eyes (average score difference = 1.4).

In human medicine, allergic conjunctivitis is concomitant with atopic dermatitis in 25–45% of adults [18] and 33% of children [19]. In our study, 94% of the cases presented with both cAC and AD symptons. The small number of cAC cases in dogs without atopic dermatitis or under remission (7 dogs out of 122) reinforces the idea that allergic conjunctivitis on its own is much less common, as already found by others [14], or it may pass unnoticed by the owners and untreated.

Interestingly, no correlation was found between ocular pruritus and head cutaneous lesional score. In fact, the severity of atopic dermatitis skin lesions was not statistically correlated with the clinical score for cAC. This absence of correlation is probably due to an individual dog-specific allergen response, some animals responding to the exposure with a predominant eye pattern and others with a predominant skin pattern due to the "pre-programming" of certain body regions to react to the presence of an allergen [4, 20].

For human allergic conjunctivitis several therapeutical proposals have emerged based on the severity of the clinical condition presented by the patient [2, 6, 7, 12, 13]. Taken this into account, we ought to try to outline therapeutical guidelines for the canine species, and we suggest considering 3 different clinical approaches.

In all grades of canine allergic conjunctivitis, allergen avoidance is recommended, whenever possible [7]. The use of cold compresses and refrigeration of any eyedrops used seems to contribute to ocular pruritus reduction [14], probably because the low temperature could favour vasoconstriction and therefore a decrease in conjunctival congestion, although its efficacy should be verified in prospective studies. Therefore, we recommend that these non-pharmacological techniques should also be used in all cAC cases.

Dogs with eye allergies undergo changes in the composition and stability of the tear film [21]. These changes include increased osmolarity [22] and a decrease in the lipid portion, which is responsible for increased tear evaporation [23, 24]. Another possibility is the existence of a deficiency in the conjunctival goblet cells due to the negative impact from chronically inflamed tissue, which would lead to mucin deficiency. Therefore, clinicians could consider the use of an eye lubricant to maintain ocular surface homeostasis and decrease adhesion of allergens that may accumulate on the ocular surface, potentiating the allergic reaction. Due to the potential role of mucin deficiency, a hyaluron-based tear supplement may be preferred over other types of lubricants. When the degree of pruritus promotes persistent behaviours that may cause injury to the eyelids or ocular surface, an elizabethan collar should be worn. In future prospective studies we should consider assessing for qualitative tear film deficiencies in canine eyes with allergic conjunctivitis.

In mild cases, in addition to the previous strategies, the use of a topical anti-allergic drug could be considered. Although antihistamines are likely to be of little or no benefit to treat chronic canine atopic dermatitis, they migh be useful for the ocular symptoms. In fact, dual-activity agents are currently considered the first line of treatment in human patients with allergic conjunctivitis [25], but antihistamines antagonists of H1 receptors or mast cell membrane stabilizers can also be prescribed [12]. Although there are no studies proving their efficacy in canine species, in clinical practice these drugs seem to show good results [14]. In cases where only the anti-allergic agents cannot control clinical signs, especially ocular pruritus, a non-steroidal topical anti-inflammatory, such as diclofenac or cetorolac, can be used. These drugs have already proven to reduce acute clinical signs in human allergic conjunctivitis [26, 27].

In cases of moderate allergic conjunctivitis, non-pharmacological measures accompanied by anti-allergic eye drops should also be applied, preferably those with dual-action, as these are the most effective in human medicine [7]. However, these animals often need topical application of a steroid anti-inflammatory drug to ensure control of clinical signs, as in humans [2], especially in periods of exacerbation of clinical signs. Topical corticosteroids should be used for short periods (maximum 2–3 weeks) and under the supervision of an ophthalmologist given their potential side effects [6, 12]. Dogs with moderate symptoms, especially if accompanied by other allergic manifestations such as atopic dermatitis, should be considered candidates for allergen immunotherapy, if the allergen is known. Immunotherapy has previously been shown to significantly reduce ocular score in atopic dogs with allergic conjunctivitis [28].

For severe cases, in addition to what has been proposed, the use of topical immunomodulators, such as topical cyclosporine A or tacrolimus, should be considered. In humans, topical cyclosporine A has been shown to significantly decrease clinical signs of severe, proliferative and corneal-involving forms of AC such as vernal keratoconjunctivitis and atopic keratoconjunctivitis. [29–31]. Tacrolimus has been shown to be more effective than cyclosporin A in managing allergic conjunctivitis, even in cases refractory to corticosteroids, in human patients with and without concomitant atopic dermatitis [32–34]. Nevertheless, a burning sensation and eye irritation can occur after its application [32–34].

Topical formulations of all selected drugs should always be preferred, the use of oral therapy being limited to refractory forms of the disease or those concomitant with other allergic manifestations that require it, such as canine atopic dermatitis.

Moderate and severe cases should ideally be referred to a veterinary dermatologist, since in most cases these animals present with concomitant atopic dermatitis, requiring treatments which may include oclacitinib maleate, lokivetmab, cyclosporine and oral corticosteroids, among others. These oral drugs seem to contribute to the improvement of cAC and can reduce the frequency for topical ocular treatments with immunomodulators [35]. On the long term, topical formulations of all selected drugs should always be preferred due to less systemic side effects.

Although the prevalence of keratitis is much lower than the other clinical signs, we found a correlation with an enhanced severity and chronicity of the ocular lesions due to cAC. Corneal involvement could compromisse vision, so these cases shoud ideally be referred to an ophthalmologist. Similarly, in humans, the more severe cases of AC develop corneal proliferative lesions such as atopic keratoconjunctivitis and vernal keratoconjunctivitis [29, 31, 31] and are treated by ophthalmologists. Our canine patients did not present with similar severe proliferative keratits forms, but they experienced a sudden relieve of their keratitis signs with the begining of anti-allergic therapeutics, which reinforces the allergic etiology of their symptons, and which does not happen in keratitis due to other causes that were ruled out such as trauma, irritation, quantitative tear film deficiencies and corneal ulceration. Of note, the keratitis grading was based on the clinical presentation seen in our study population and does not resemble scoring criteria from other studies; here, severe keratitis (grade 3) was positively associated with the more severe forms of cAC and generally implied corneal neovascularization, corneal fibrosis ± corneal dystrophy.

Proliferative conjunctival follicles (especially in ventral conjunctival fornix, anterior and posterior surface of the third eyelid) can be observed in canine eyes with chronic ocular inflammation, although this hiperplasia of the conjunctival lynphoid tissue is not characterisitic for allergic reactions in the authors´experience. Generally observed in young dogs who are still mounting their immune response to external allergens, proliferative conjunctival follicles are not common in older dogs with chronically inflamed eyes. Nevertheless, the presence of proliferative conjunctival follicles can also be compatible with allergic conjunctivitis and this feature should be taken into consideration in future studies.

One of the limitations of this study lies in the fact that the classification of the grade of severity of each clinical sign implies a certain degree of subjectivity between observers. Our results are subjective due to the retrospective nature of the study and lack of standardized scoring for this ocular disease in dogs, and may have been under- or overestimated in some cases. Future work should focus on a simplified grading scheme that is reproducible and clinically useful, as exemplified by scoring systems reported by Eaton et al. and Sebbag et al. [36, 37], ideally correlating with the different therapeutical protocols and this grading scheme could be used by multiple evaluators for additional validation.

Also, the location of conjunctival hyperemia/chemosis should be considered (bulbar vs. palpebral conjunctiva, dorsal vs. ventral conjunctiva), since an allergic reaction is often most pronounced in the ventral conjunctiva and third eyelid, where most allergens accumulate, which can help clinicians differentiate cAC from other conjunctivitis causes that usually present with more diffuse conjunctival inflammation.

It would have been ideal to use only one CADESI scale in this paper (either CADESI-03 or CADESI-04), since they have different graduations. Neverthless the reasons why both were used regards the time frame of the study (initially CADESI 04 was not available as this study began in 2013 and this version was only available in 2014), the retrospective nature of the study and the fact that patients were scrutinized for other projects while the last version of the scale was being used. Additionally, it is not doable to convert CADESI-04 in CADESI-03. The earlier vertion (CADESI-03) evaluates 62 body regions, has self-induced alopecia as a fourth lesion and a severity scale form of six grades. Conversely, CADESI 04 is a validated simplification of CADESI 03, with only 20 tipically affected body sites, three lesions (erythema, liquenification and alopecia/excoriation) and has a severity scale of four grades. Therefore, to allow for data uniformization, all CADESI quantitative values were converted into a qualitative scale (mild, moderate, severe or in remission) according to pre-established categorization.

## Conclusions

We aim to contribute to disease characterization and clinical staging in an attempt to address the lack of established criteria in dogs with allergic conjunctivitis. Although the suggested clinical score and therapeutic guidelines still require validation with prospective clinical studies, this proposal may constitute the initial kick off to foment more studies in cAC diagnostic and therapeutical approaches.

Diagnostic standardization will allow for a better clinical monitorization, with the ultimate goal of assisting veterinarians with clinical diagnosis and therapeutics, improving confort and well being of our allergic patients.

## Data Availability

The datasets analysed during the current study are not publicly available due to data protection but all the relevant information concerning the patients data are included in the manuscript and will be available from the corresponding author on reasonable request.
